# Chitin and Chitosan as Multipurpose Natural Polymers for Groundwater Arsenic Removal and As_2_O_3_ Delivery in Tumor Therapy

**DOI:** 10.3390/md8051518

**Published:** 2010-04-28

**Authors:** Letizia Da Sacco, Andrea Masotti

**Affiliations:** Gene Expression–Microarrays Laboratory, IRCCS-Children’s Hospital Bambino Gesù, P.za S.Onofrio 4, 00165, Rome, Italy; E-Mail: letizia.dasacco@opbg.net

**Keywords:** chitin, chitosan, arsenic remediation, arsenic trioxide, tumor therapy

## Abstract

Chitin and chitosan are natural polysaccharide polymers. These polymers have been used in several agricultural, food protection and nutraceutical applications. Moreover, chitin and chitosan have been also used in biomedical and biotechnological applications as drug delivery systems or in pharmaceutical formulations. So far, there are only few studies dealing with arsenic (As) removal from groundwater using chitin or chitosan and no evidence of the use of these natural polymers for arsenic trioxide (As_2_O_3_) delivery in tumor therapy. Here we suggest that chitin and/or chitosan might have the right properties to be employed as efficient polymers for such applications. Besides, nanotechnology offers suitable tools for the fabrication of novel nanostructured materials of natural origin. Since different nanostructured materials have already been employed successfully in various multidisciplinary fields, we expect that the integration of nanotechnology and natural polymer chemistry will further lead to innovative applications for environment and medicine.

## 1. Introduction

Chitin is an abundant natural polysaccharide produced by arthropods and crustaceans. Chitin is found in diatoms, nematodes, molluscs and as a structural polysaccharide in basidiomycetes and filamentous fungal cell walls, constituting almost 16% of the dry weight of the organism [1]. Chitin deacetylation leads to the formation of chitosan. The process involves the use of strong alkali solutions for the removal of *N*-acetyl groups both at room or elevated temperatures [2,3]. Chitin and chitosan are suitable functional materials due to their excellent properties such as biocompatibility, biodegradability, non-toxicity and adsorption properties, to cite only a few [4]. Chitin and chitosan are highly basic polysaccharides with unique properties like the ability to form films [5] to react with polyanions [6,7] as well as to chelate and remove metal ions [8]. A wide variety of medical applications for chitin and chitosan derivatives have been reported in the last few years [9–11].

However, the versatitily of chitin and chitosan was emphasized in two recent, and somehow controversial, applications in the field of arsenic chemistry. In fact, the potential of chitin and chitosan can be exploited either for inorganic arsenic removal in groundwater or in the design of novel delivery vectors for arsenic trioxide (As_2_O_3_) in tumor therapy. While a high dose of inorganic arsenic (iAs) is toxic for individuals, As_2_O_3_ is used as an antineoplastic agent in most common malignancies. We think that this interesting paradoxical behavior merits a deeper investigation in the near future by investigators and clinicians involved in these two different research fields.

## 2. Chitin and Chitosan for Arsenic Species Complexation and Removal

Arsenic (As) is a metalloid that occurs naturally in the environment and is present in soil, groundwater and plants [12]. Inorganic arsenic (iAs) is the most toxic form, and has been classified in group 1 as carcinogenic to humans by the International Agency for Research on Cancer [13]. In fact, arsenic may induce lung, urinary bladder and primary skin cancer.

In China, India and Bangladesh, As contamination ([As] > 100.000 μg/L) of groundwater used for drinking is a serious threat for human health [14]. The WHO provisional guideline value for arsenic in drinking water has been set to 10 μg/L [15]. Moreover, some cereal grains and cereal-based products (*i.e.*, rice) can be contaminated by a high amount of arsenic. Besides, the influence of processing and cooking of these foods should be taken into account if iAs-contaminated water is used [16,17]. The need to regulate As exposure in infants consuming rice-based products has been recently highlighted by us since the European Union has not fixed a maximum tolerable limit for these foods so far [18].

Therefore, the identification and use of low-cost methods and safe materials to remove As from drinking water is highly desirable [19]. Several methods are available for the removal of As and have been reviewed by Mohan *et al.* [20]. Among them, we mention co-precipitation, flotation, ion exchange, ultrafiltration, and reverse osmosis. Arsenic adsorption from aqueous solution has received more attention due to its higher efficiency. Activated alumina [21], activated carbon [22] and bauxsol [23] have been used as efficient adsorbents. Even goethite and hematite [24], iron oxide-coated sand [25], ferrihydrite [26], and Fe(III)-loaded resins [27] have been employed because of the selectivity and affinity of Fe(III) toward iAs species. Although these resins are efficient and selective in removing As, their applicability is limited due to their high cost. For these reasons, adsorption of arsenic using natural products has emerged as a viable option [28,29]. Chitin and chitosan have been reported to be efficient heavy metal scavengers due to the presence of hydroxyl and amino groups [30]. In a recent study by Sun *et al.* [31] it was observed that sulfur atoms also have a strong affinity for arsenic. Therefore, the authors prepared polyaspartate and chitosan blends derivatized with -SH functionalities. Blends were reacted with mercaptoacetic (thioglycolic) acid affording the –SH derivative. This polymer showed better arsenic-removal behavior (As removal > 22%) than other adsorbents, leading to a lower arsenic equilibrium concentration.

Removal of arsenic from contaminated drinking water was also studied on a chitosan/chitin mixture that showed a capacity of 0.13 μ-equivalents As/g (pH = 7.0) [32].

Recently, removal of both As(III) and As(V) by chitosan-coated alumina or molybdate-impregnated chitosan was reported [33,34]. These methods show a very high adsorption capacity at pH = 4 (56.50 and 96.46 mg/g for As(III) and As(V), respectively), but other interfering ions (*i.e.*, phosphate) may limit or abolish the efficiency of this adsorbent.

Chitin and chitosan were comparatively employed for remediation of chromated copper arsenate (CCA) preservative-treated wood in recent years due to release of chromium, copper, and arsenic elements from waste wood during land filling, burning, composting, and other disposal methods [35]. Limiting the analysis to As species only, chitin removed 63% of As from treated sawdust, while using the same amount of chitosan the recovery was only 30%. These results clearly show that even this acetylated polysaccharide can be efficiently employed for arsenic removal. However, chitosan is generally preferred over chitin in the vast majority of applications [36,37].

## 3. Chitosan for As_2_O_3_ Incorporation and Delivery

In a completely different field, arsenicals and compounds like arsenic trioxide (As_2_O_3_) have been used therapeutically in traditional Chinese medicine for a long time. Arsenic trioxide administered intravenously was reintroduced in the year 2000 by the US Food and Drug Administration as an anticancer agent for the treatment of acute promyelocytic leukemia (APL), even for patients with relapsed or refractory forms [38].

It was hypothesized that the anticancer mechanisms of As_2_O_3_ may be due not only to the induction of apoptosis as previously reported by Zhang [39], but also by reactivation of silenced tumor suppressor genes through DNA demethylation [40].

Other studies reviewed by Cui *et al.* investigated the use of As_2_O_3_ in the treatment of solid tumors, including liver, prostate, neuroblastoma, head and neck, gastric, transitional cell, and renal cell carcinoma, esophageal, prostate, colorectal cancer, fibroblastoma, and melanoma [40].

However, the efficacy of As_2_O_3_ against human solid tumors is poor, even at a high As dosage. Consequently, different strategies aimed at increasing its efficacy such as local delivery (intratumoral injection) have been devised [41]. This strategy not only increases the localization of specific drugs in the tumor, but also reduces the unspecific uptake by other organs (targeted delivery) and presents an anti-invasive activity [42].

The incorporation of As_2_O_3_ with chitosan nanospheres to form novel arsenic-containing chitosan-based nanocarriers represents an innovative strategy that might be exploited. Additionally, chitosan itself has been claimed to show antineoplastic activities tested both *in vitro* and *in vivo* [43] even if the results are still controversial [44]. These nanovectors, as other common nanomaterials, can be potentially used for their ability to exploit the Enhanced Permeability and Retention (EPR) effect already used for the delivery of small molecules [45,46]. The EPR effect is based on the fact that tumor cells grow quickly and stimulate the production of blood vessels for nutrition and oxygen supply. These newly formed tumor vessels are usually abnormal in form and architecture. They present wide fenestrations where engineered systems may enter and deliver their cargo. Therefore, such delivery vehicles have the property to accumulate much more in tumor tissues than in normal tissues just exploiting their dimensions. To achieve the EPR effect, As_2_O_3_ can be therefore incorporated into chitosan nanospheres, which might assure a quite prolonged diffusion, biocompatibility and safety, while exploiting the EPR effect. An already reported example is represented by chitosan nanospheres [47]. These nanospheres have been studied for their efficient DNA incorporation and delivery properties.

Alternatively, due to the versatility of chitosan to give larger biocompatible microspheres, As_2_O_3_ can be incorporated into larger microspheres, as already achieved with DNA, using various fabrication methods [48]. Size and shape of these systems are important factors for several medical applications: to improve bioavailability (*i.e.*, overcoming enzymatic or adsorption barriers and in the case of nasal administration the mucociliary clearance) and to prolong the residence time of drug delivery systems at the site of drug absorption [49].

## 4. Interactions of Arsenite, Arsenate and Arsenic Trioxide (As_2_O_3_) with Chitosan

It is generally believed that electrostatic interactions, metal chelation and ion pairs formations are the three main mechanisms hypothesized to occur when a metal is adsorbed by chitosan [30,50]. Surface adsorption, chemi- and physisorption, diffusion and adsorption-complexation mechanisms may also occur [51] as a consequence of ion-exchange, hydrogen bonds, hydrophobic and van der Waals interactions.

The occurrence of interactions between chitosan and anionic molecules (dyes) was studied by thermodynamic methods recognizing that chemisorption (ion-exchange, electrostatic attractions) is the most prevalent mechanism with the pH as the main factor affecting adsorption [52,53].

**Figure 1:**
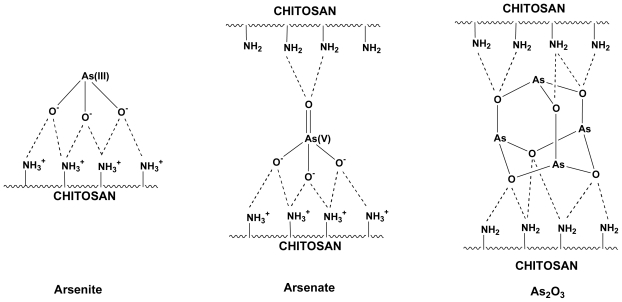
A model of interaction between chitosan and As(III) (arsenite), As(V) (arsenate) species and As_2_O_3_ oxide.

We hypothesized a model of interaction between chitosan and As(III) (arsenite)/As(V) (arsenate) species or arsenic oxide As_2_O_3_ that we have presented in Figure 1. The arsenite anion may preferentially form electrostatic interactions with protonated chitosan amino groups. Arsenate may instead interact with the polymer through electrostatic interactions and hydrogen bonds. For what concerns the arsenic oxide As_2_O_3_, the interaction might occur mainly through hydrogen bonds.

## 5. Conclusions

From all the studies appearing in the literature over the last few years, it appears clear that the use of chitin and chitosan for toxic inorganic As removal or As_2_O_3_ delivery for tumor therapy is quite a neglected topic. We think that nanotechnology and biomedicine applied to these two aspects would help to solve not only some environmental pollution aspects, but also to improve the life quality of future generations and to devise novel prevention strategies and innovative therapeutic approaches.
